# Diagnostic accuracy of fibrosis tests in children with non‐alcoholic fatty liver disease: A systematic review

**DOI:** 10.1111/liv.14908

**Published:** 2021-05-11

**Authors:** Laura G. Draijer, Janneke P. M. van Oosterhout, Yasaman Vali, Sabrina Zwetsloot, Johanna H. van der Lee, Faridi S. van Etten‐Jamaludin, Malika Chegary, Marc A. Benninga, Bart G. P. Koot

**Affiliations:** ^1^ Department of Pediatric Gastroenterology and Nutrition Amsterdam University Medical Centers Academic Medical Center Emma Children's Hospital University of Amsterdam Amsterdam The Netherlands; ^2^ Amsterdam Reproduction & Development Research Institute Amsterdam University Medical Centers Academic Medical Center Emma Children's Hospital Amsterdam The Netherlands; ^3^ Amsterdam UMC University of Amsterdam Gastroenterology and Hepatology Amsterdam Gastroenterology Endocrinology Metabolism Research Institute Amsterdam Netherlands; ^4^ Department of Epidemiology and Data Science Amsterdam University Medical Centers Academic Medical Center University of Amsterdam Amsterdam The Netherlands; ^5^ Paediatric Clinical Research Office Amsterdam University Medical Centers Academic Medical Center/Emma Children's Hospital University of Amsterdam Amsterdam The Netherlands; ^6^ Knowledge Institute of the Dutch Federation of Medical Specialists Utrecht The Netherlands; ^7^ Amsterdam UMC University of Amsterdam Research Support Medical Library AMC Amsterdam The Netherlands; ^8^ Department of Paediatrics Onze Lieve Vrouwe Gasthuis Hospital Amsterdam The Netherlands

**Keywords:** diagnosis, hepatic fibrosis, obesity, paediatric

## Abstract

**Background & Aims:**

Non‐alcoholic fatty liver disease (NAFLD) has become the most common chronic liver disease in children. Even at young age, it can progress to liver fibrosis. Given the drawbacks of liver biopsy, there is a need for non‐invasive methods to accurately stage liver fibrosis in this age group. In this systematic review, we evaluate the diagnostic accuracy of non‐invasive methods for staging liver fibrosis in children with NAFLD.

**Methods:**

We searched MEDLINE, Embase, Web of Science and the Cochrane Library, for studies that evaluated the performance of a blood‐based biomarker, prediction score or imaging technique in staging liver fibrosis in children with NAFLD, using liver biopsy as the reference standard.

**Results:**

Twenty studies with a total of 1787 NAFLD subjects were included, which evaluated three prediction scores, five simple biomarkers, two combined biomarkers and six imaging techniques. Most studies lacked validation. Substantial heterogeneity of studies and limited available study data precluded a meta‐analysis of the few fibrosis tests evaluated in more than one study. The most consistent accuracy data were found for transient elastography by FibroScan®, ELF test and ultrasound elastography, with an area under the receiver operating characteristics curve varying between 0.92 and 1.00 for detecting significant fibrosis.

**Conclusion:**

Due to the lack of validation, the accuracy and clinical utility of non‐invasive fibrosis tests in children with NAFLD remains uncertain. As studies have solely been performed in tertiary care settings, accuracy data cannot directly be translated to screening populations.

**Abbreviations**
ALT: alanine aminotransferase
AUC: area under the receiver operating characteristics curve
CK‐18: cytokeratin‐18
ELF test: enhanced liver fibrosis test
GGT: gamma glutamyl transferase
HA: hyaluronic acid
LR−: negative likelihood ratio
LR+: positive likelihood ratio
MCP‐1: monocyte chemoattractant protein 1
MRE: magnetic resonance elastography
NAFLD: non‐alcoholic fatty liver disease
NASH CRN: NASH Clinical Research Network
NASH: non‐alcoholic steatohepatitis
NPV: negative predictive value
PAI‐1: plasminogen activator inhibitor 1
PIIINP: procollagen type III amino terminal peptide
PNFI: paediatric NAFLD fibrosis index
PPV: positive predictive value
QUADAS‐2: quality Assessment of Diagnostic Accuracy Studies 2
SWE: shear wave elastography
TE: transient elastography

**Key points**

- The 16 included diagnostic tests for detecting fibrosis in children with non‐alcoholic fatty liver disease were mostly evaluated in small studies and lacked validation.
- The most consistent data showing good accuracy were found for FibroScan®, ELF test and ultrasound elastography.
- Interpretation of results of non‐invasive methods to stage liver fibrosis in children remains cumbersome due to the lack of well validated accuracy data.

## 1 INTRODUCTION

Due to the obesity epidemic, non‐alcoholic fatty liver disease (NAFLD) has become the most common chronic liver disease in children and adults.^1^ The pooled prevalence of NAFLD in children with obesity is 34% (95% CI: 27.8% to 41.2%).^2^ Simple steatosis, or non‐alcoholic fatty liver (NAFL), is the first stage of the NAFLD spectrum and is defined as fat accumulation in more than 5% of the hepatocytes in the biopsy specimen on histological evaluation. A NAFLD subtype that is characterized by significant inflammation is categorized as non‐alcoholic steatohepatitis (NASH) and can progress to severe stages of fibrosis and cirrhosis.^1^ Although most children with NAFLD will have simple steatosis, advanced fibrosis is reported in up to 17% of children referred to liver centres after screening,^3^, ^4^ and some cases of NAFLD‐related cirrhosis in children have been reported.^5^, ^6^ Evidence shows that fibrosis is the most important predictor for liver‐related complications in adults, such as liver failure and hepatocellular carcinoma, and is associated with increased overall mortality.^7^, ^8^ Therefore, liver fibrosis represents the most clinically relevant determinant of long‐term outcomes in this disorder.^7^ The development of fibrosis at a young age is considered worrisome and, although long‐term longitudinal studies are lacking to prove this, could be related to a higher risk of developing long‐term liver and non‐liver complications. Current paediatric guidelines recommend screening for fibrosis in children with NAFLD but do not specify what test should be used to assess fibrosis.^1^, ^9^ In addition, accurate tests could serve as surrogate endpoints in future paediatric therapeutic trials.^10^

Liver biopsy is the current reference standard to determine the stage of liver fibrosis in patients with NAFLD. However, in addition to the risk of complications, the costly and invasive nature of this procedure makes it unsuitable for screening purposes or for monitoring disease progression in this highly prevalent disorder.^11^ Moreover, the diagnostic accuracy of liver biopsy is not optimal due to sampling variability caused by the often patchy distribution of NAFLD in the liver and interobserver and intraobserver variability of the histological interpretation.^12^ Therefore, there is an urgent need for accurate, safe and cost‐effective alternatives to accurately stage liver fibrosis in patients with NAFLD. Over the past decade, many fibrosis tests have been developed, ranging from simple laboratory tests to more complex biomarkers or prediction scores as well as imaging techniques.^13^ Although most of these tests were developed and validated in the adult population, several research groups have investigated their utility in the paediatric population.^14^ This systematic review aims to appraise the diagnostic accuracy of non‐invasive methods for detecting and staging liver fibrosis in children with NAFLD.

## 2 METHODS

### 2.1 Literature search strategy

A sensitive search strategy was developed in collaboration with an experienced medical librarian and conducted in PubMed/MEDLINE, Ovid/EMBASE, Web of Science and Cochrane Library (Data S1 and S2). The search comprised the following search terms: Non‐alcoholic Fatty Liver Disease, children, diagnosis and fibrosis. No date limit was applied to the search. The bibliographic reference lists of included articles and reviews were manually searched. Article selection was accomplished in April 2020.

### 2.2 Selection criteria

Articles were included if they fulfilled the following criteria: (a) the study included patients with biopsy proven NAFLD/NASH/steatosis, and in case of inclusion of other causes of chronic liver disease, the study provided discrete data on the NAFLD population separately; (b) the study consisted of children up to 18 years, or reported separately on children, if adults were included; (c) the study evaluated the performance of a blood‐based biomarker, prediction score or imaging technique to detect different stages of liver fibrosis; (d) liver biopsy was used as the reference test; (e) the study included ≥60 participants or the diagnostic test was reported in ≥2 studies; and (f) the study provided enough data to construct a 2 × 2 table. Studies were excluded if they did not meet the inclusion criteria or (a) had a case report, case series, conference abstract or commentary design and (b) were conducted in animal subjects. No language restriction was used.

### 2.3 Data extraction and quality assessment

Two authors (L.D. and J.O.) independently screened the titles and abstracts to identify articles that met the inclusion criteria using Rayyan software (https://rayyan.qcri.org). Then, the full texts of the potentially eligible studies were screened independently by the two authors. Data extraction was performed independently by two authors (L.D. and S.Z.) using a predesigned data extraction form. For studies that included adults or patients with various liver diseases as well, that did not report paediatric data or NAFLD data separately, the authors were contacted and requested to provide raw data. The study design, patients characteristics, histological scoring system that was used for fibrosis staging, prevalence of different fibrosis stages and accuracy data of different diagnostic methods (thresholds, number of true positives [TP], false positives [FP], true negatives [TN], false negatives [FN], sensitivity, specificity, positive predictive value [PPV], negative predictive value [NPV] and [if provided] the area under the receiver operating characteristics curve [AUC]) were extracted from each included article. For uniformity, the fibrosis stages assessed by a histological scoring system other than NASH Clinical Research Network (CRN) were converted to fibrosis stages according to NASH CRN.^15^ The different fibrosis scoring systems are presented in Data S3. The Quality Assessment of Diagnostic Accuracy Studies 2 (QUADAS‐2) tool was used to evaluate the methodological quality of the included studies. Any disagreement between the two authors (L.D. and J.O.) was resolved through discussion. A third reviewer (B.K.) was consulted when necessary.

### 2.4 Data analysis

Medcalc was used to analyse the tests for sensitivity, specificity, PPV, NPV, positive likelihood ratio (LR+) and negative likelihood ratio (LR−).^16^ Review Manager version 5.3 was used for quality assessment and creating figures. A meta‐analysis of any of the evaluated fibrosis tests could not be performed because a summary ROC curve (HSROC) and summary sensitivities and specificities could not be constructed due to the use of different reported thresholds and different settings of magnetic resonance elastography (MRE) and ultrasound elastography among studies.

## 3 RESULTS

### 3.1 Search results

**FIGURE 1:**
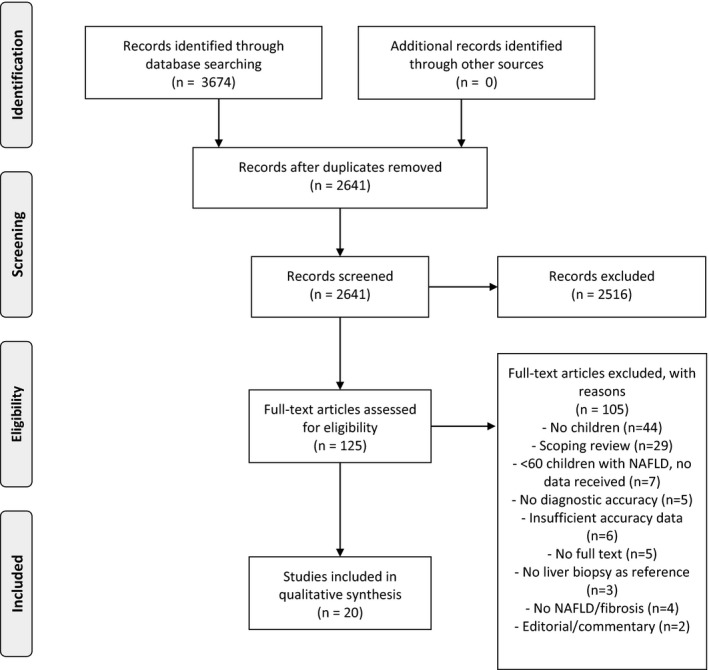
PRISMA flow diagram of primary studies

A total of 3674 records were retrieved from our search. After removing duplicates, 2641 records were retained. After screening the titles and abstracts, full text of 125 articles were reviewed of which 20 studies met our inclusion criteria. Reasons for exclusion of the 105 records are shown in Figure 1. Six of the excluded studies did not report sufficient data to create 2 × 2 tables but reported the AUCs. Data S4 shows the AUCs of these six excluded studies and of four included studies that, in addition to tests with data to create a 2 × 2 table, reported AUCs of different thresholds or various types of tests. The included studies evaluated three prediction scores, five simple biomarkers, two combined biomarkers and six imaging techniques.

### 3.2 Study characteristics

Characteristics of the 20 included studies are provided in Table 1. All studies were performed in tertiary hospitals of which eight were paediatric liver transplant centres. All studies had a cross‐sectional design. Sixteen studies used prospectively collected data, one study used retrospectively collected data and in three studies, this was not specified. Thirteen studies reported on the accuracy of detecting mild fibrosis (≥F1),^17^, ^18^, ^19^, ^20^, ^21^, ^22^, ^23^, ^24^, ^25^, ^26^, ^27^, ^28^ 10 studies on detecting significant fibrosis (≥F2)^19^, ^20^, ^24^, ^25^, ^29^, ^30^, ^31^, ^32^, ^33^, ^34^ and nine studies on detecting advanced fibrosis (≥F3).^19^, ^20^, ^24^, ^28^, ^30^, ^31^, ^35^, ^36^, ^37^ The time interval between the index test and liver biopsy varied between 0 days and 6 months.

**TABLE 1:** Study characteristics

Author, year	Country, hospital	Setting	Inclusion period	Population	Histological scoring system	Time interval^b^	Index test(s)	N^a^ (total = 1787)	Target condition(s)/N (%)	Male, n (%)	Mean age, years (SD)	Mean BMI *z* score (SD) or BMI percentile (SD)	Mean ALT, IU/L (SD)	Mean HOMA‐IR (SD)
Fitzpatrick, 2012	UK, London	Tertiary Hospital/Liver Transplant Centre	NR	Biopsy proven NAFLD	NASH CRN	NR	‐ PAI‐1 ‐ MCP‐1	40	≥F3/13 (33)	30 (75)	13.4 (11.5‐15.2)^c^	2.12 (1.77‐2.31)^c^	67 (50‐83)^c^	4.08 (2.18‐5.89)^c^
Fitzpatrick, 2010	UK, London	Tertiary Hospital/Liver Transplant Centre	NR	Biopsy proven NAFLD	NASH CRN	Within 3 months	CK‐18 (M30)	45	≥F2/23 (51)	25 (57)	12.7 (10.2‐14.3)^c^	1.7 (1.2‐2.1)^c^	61 (23‐178)^c^ for <F2 and 109 (92‐136) for ≥F2	0.68 (0.55‐0.94)^c^ for <F2 and 0.94 (0.56‐1.96) for ≥F2
Mandelia, 2016	Italy, Rome	Tertiary Hospital/Liver Transplant Centre	2008‐2011	Biopsy proven NAFLD	NASH CRN	0 days	CK‐18 (M30)	201	≥F1/136 (68)	74 (37)	10.7 (2.5)	97 (93‐98)^c^	70 (50‐87)^c^	2.6 (1.7)
Lebensztejn, 2011	Poland, Bialystok	Tertiary Hospital	NR	Biopsy proven NAFLD	Brunt & Kleiner	1 day	‐ HA ‐ CK‐18 (M30)	52	≥F1/19 (37)	39 (75)	12.1 (10.5‐14.4.)^c^	NR	70 (51‐114)^c^	1.9 (0‐3.7)^c^
Nobili, 2010	Italy, Rome	Tertiary Hospital/Liver Transplant Centre	2006‐2009	Biopsy proven NAFLD	NASH CRN	0 days	HA	100	≥F1/65 (65) ≥F2/15 (15)	68 (68)	NR	NR	NR	NR
Mosca, 2019	Italy, Rome	Tertiary Hospital	2015‐2018	Biopsy proven NAFLD	NASH CRN	NR	PIIINP	204	≥F2/45 (22) ≥F3/7 (3)	154 (76)	13.1 (2.2)	NR	28 (19‐46)^c^ for non‐NASH, 33 (23‐56) for NASH	3.6 (2.1‐4.4)^c^ for non‐NASH, 4.1 (2.7‐6.1) for NASH
Alkhouri, 2011	Italy, Rome	Tertiary Hospital/Liver Transplant Centre	2007‐2009	Biopsy proven NAFLD	NASH CRN	NR	‐ ELF test (Guha algorithm) ‐ PNFI	111	≥F1/76 (68)	73 (67)	10.5 (9.5‐11.4)^c^	97 (93‐98)^c^	67 (45‐89)^c^	2.4 (1.6‐3.6)^c^
Nobili, 2009	Italy, Rome	Tertiary Hospital/Liver Transplant Centre	2004‐2006	Biopsy proven NAFLD	Brunt & Kleiner	3 days	ELF test (Guha algorithm)	112	≥F1/75 (67) ≥F2/17 (15) ≥F3/8 (7)	64 (57)	13.8 (3.3)	1.8 (0.6)	80 (63)	2.5 (1.1)
Alkhouri, 2014	Italy, Rome	Tertiary Hospital/Liver Transplant Centre	NR	Biopsy proven NAFLD	NASH CRN	1 week	PNFS	242	≥F3/36 (17)	90 (37)	12.4 (3.1)	93 (13)	75 (55‐99)^c^	2.2 (1.4‐3.2)^c^
Nobili, 2009	Italy, Rome	Tertiary Hospital/Liver Transplant Centre	2004‐2008	Biopsy proven NAFLD	NASH CRN	NR	PNFI	203	≥F1/141 (69)	136 (67)	11.9 (2.8)	1.8 (0.7)	67 (61)	2.4 (1.9)
Alkhouri, 2013	Italy, Rome	Tertiary Hospital/Liver Transplant Centre	NR	Biopsy proven NAFLD	NASH CRN	NR	‐ PNFI ‐ TE (FibroScan S‐probe®)	67	≥F2/10 (15)	46 (69)	8.5 (2.4)	NR	83 (55)	NR
Lee, 2013	USA, Boston	Tertiary Hospital/Liver Transplant Centre	2006‐2011	Various liver diseases	METAVIR	Within 6 months	‐ TE (FibroScan® M‐probe) ‐ HA	10	≥F3/4 (40)	5 (50)	13.9 (2.4)	2.12 (0.40)	NR	NR
Nobili, 2008	Italy, Rome	Tertiary Hospital/Liver Transplant Centre	2007‐2008	Biopsy proven NASH	Brunt & Kleiner	Within 6 months	TE (FibroScan® M‐probe)	50	≥F1/39 (78) ≥F2/12 (24)	≥F3/5 (10)	31 (62)	13.1 (2.0)	NR	72 (38)
Hudert, 2018	Germany, Berlin	Tertiary Hospital	2014‐2017	Suspected NASH	NASH CRN	NR	Time‐harmonic elastography	67	≥F1/48 (72) ≥F2/31 (46) ≥F3/18 (27)	51 (76)	14.1 (2.2)	2.8 (0.6)	108 (70)	6.9 (3.7)
Phelps, 2016	USA, San Francisco	Tertiary Hospital	2014‐2015	Various liver diseases	METAVIR and NASH CRN	0 days	Point SWE (Siemens Acuson S3000)	5	≥F1/4 (80)	3 (60)	11.2 (2.7)	NR	154 (93)	NR
Garcovich, 2017	Italy, Rome	Tertiary Hospital/Liver Transplant Centre	2015	Biopsy proven NASH	Brunt	Within 6 months	SWE (Aixplorer US system)	68	≥F2/16 (24)	37 (54)	12.6 (2.5)	NR	41 (26)	4.5 (2.1)
Farmakis, 2019	USA, St. Louis	Tertiary Hospital	2015‐2018	Various liver diseases	METAVIR, Ishak and NASH CRN	Within 1 month	2D‐SWE (GE LOGIQ E9 system)	33	≥F1/26 (79)	24 (73)	13.3 (3.4)	NR	150 (104)	NR
Schwimmer, 2017	USA, San Diego and Texas	Tertiary Hospital	NR	Biopsy proven NAFLD	NASH CRN	Within 6 months	2D MR Elastography	90	≥F1/36 (40) ≥F3/6 (7)	66 (73)	13.1 (2.4)	2.1 (0.3)	NR	NR
Trout, 2018	USA, Cincinnati	Tertiary Hospital	2012‐2016	Various liver diseases	NASH CRN	Within 3 months	MR Elastography	44	≥F2/23 (52)	NR	NR	NR	NR	NR
Hudert, 2019	Germany, Berlin	Tertiary Hospital	2014‐2016	Obese/overweight suspected NASH	NASH CRN	NR	Multifrequency MRE	50	≥F1/35 (70) ≥F2/23 (46) ≥F3/14 (28)	40 (80)	14.1 (2.1)	2.7 (0.6)	109 (67)	6.5 (3.1)

Abbreviations: ALT, alanine aminotransferase; BMI, body mass index; CK‐18, cytokeratin 18; ELF test, enhanced liver fibrosis test; HA, hyaluronic acid; HOMA‐IR, homeostatic model assessment for insulin resistance; MCP‐1, monocyte chemoattractant protein 1; MRE, magnetic resonance elastography; NAFLD, non‐alcoholic fatty liver disease; NASH CRN, non‐alcoholic steatohepatitis Clinical Research Network; NR, not reported; PAI, plasminogen activator inhibitor 1; PIIINP, procollagen type III amino terminal peptide; PNFI, paediatric NAFLD fibrosis index; PNFS, paediatric NAFLD fibrosis score; SWE, shear wave elastography; TE, transient elastography.

^a^ Number of patients with NAFLD.

^b^ Time interval between liver biopsy and index test.

^c^ Median with IQR.

### 3.3 Patient characteristics

In total, 1787 subjects with NAFLD were included. The mean age of the NAFLD patients ranged from 8.5 to 14.1 years and 64% were male (range 24% to 80%). The prevalence ranged from 37% to 98% per study for ≥F1,^17^, ^18^, ^19^, ^20^, ^21^, ^22^, ^23^, ^24^, ^25^, ^26^, ^27^, ^28^, ^29^, ^30^, ^32^, ^36^, ^37^ 10% to 48% for ≥F2^17^, ^19^, ^20^, ^21^, ^22^, ^24^, ^25^, ^26^, ^27^, ^28^, ^29^, ^30^, ^31^, ^32^, ^33^, ^34^, ^36^ and 3% to 40% for ≥F3,^17^, ^18^, ^19^, ^20^, ^21^, ^22^, ^24^, ^25^, ^26^, ^27^, ^28^, ^29^, ^30^, ^31^, ^35^, ^36^, ^37^ based on the 17 studies that reported separate prevalence data.

### 3.4 Methodological quality assessment

**FIGURE 2:**
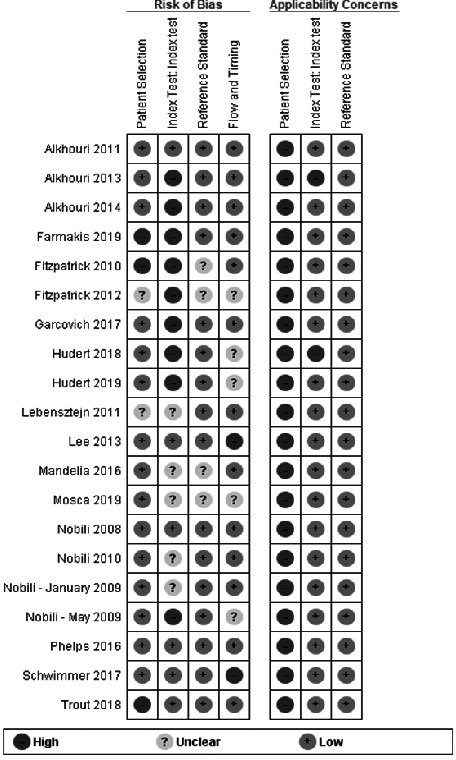
Quality assessment per study

**FIGURE 3:**
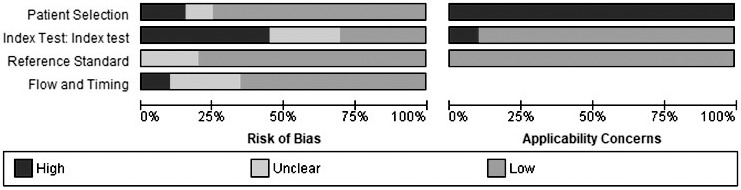
Summary of quality assessment

The results of the methodological quality assessment of the individual studies using the QUADAS‐2 tool are presented in Figure 2 and are summarized in Figure 3. Only three studies had a low risk of bias in all four domains. In all studies, there were concerns about applicability regarding patient selection because patients were recruited in tertiary hospitals and were selected to undergo liver biopsy based on clinical grounds. In two studies, there were concerns about applicability regarding the index test. These tests evaluated time‐harmonic elastography: a technique developed by Hudert et al.^19^ which is not commercially available and the S‐probe of the FibroScan® (Echosens, France) that was evaluated in children with overweight/obesity by Alkhouri et al.^34^ This specific probe was developed for children with a chest circumference < 75 cm and is generally unsuitable for children with obesity.

### 3.5 Detecting mild fibrosis (≥F1)

Thirteen studies investigated non‐invasive methods to detect mild fibrosis (≥F1). These studies addressed prediction scores, that is, cytokeratin‐18 (CK‐18) combined with waist circumference and the paediatric NAFLD fibrosis index (PNFI), which is based on age, waist circumference and triglycerides. The evaluated biomarkers were the enhanced liver fibrosis (ELF) test, hyaluronic acid (HA), CK‐18 and the combination of CK‐18 and HA. The imaging techniques evaluated were MRE, transient elastography (TE) of FibroScan®, shear wave elastography (SWE), time‐harmonic elastography and point shear wave elastography. The results of these 13 studies are presented in Table 2.

**TABLE 2:** Studies on detecting mild fibrosis

Study	Test	AUC (95% CI)	Threshold	TP	FP	FN	TN	Sensitivity (95% CI)	Specificity (95% CI)	PPV (95% CI)	NPV (95% CI)
Prediction scores											
Nobili, 2009	PNFI	0.85 (0.80‐0.90)	≥1	140	58	1	4	96 (91‐98)	29 (18‐42)	75 (68‐81)	75 (53‐90)
			≥2	137	53	5	9	97 (93‐99)	145 (7‐26)	72 (65‐78)	69 (39‐91)
			≥3	135	44	6	18	96 (91‐98)	29 (18‐42)	75 (68‐82)	75 (53‐90)
			≥4	132	35	9	27	94 (88‐97)	44 (31‐57)	79 (72‐85)	75 (58‐88)
			≥5	125	29	16	33	88 (82‐93)	53 (40‐66)	81 (74‐87)	67 (53‐80)
			≥6	115	23	26	39	82 (74‐88)	63 (50‐75)	83 (76‐89)	60 (47‐72)
			≥7	108	10	33	52	77 (69‐83)	84 (72‐92)	92 (85‐96)	61 (50‐72)
			≥8	88	6	53	56	62 (54‐70)	90 (80‐96)	94 (87‐98)	51 (42‐61)
			≥9	65	1	76	61	46 (38‐55)	98 (91‐100)	99 (92‐100)	45 (36‐53)
Alkhouri, 2011	PNFI	0.761 (0.661‐0.861)	≥3.47^a^	71	24	5	11	93 (85‐98)	31 (17‐49)	75 (70‐79)	69 (45‐85)
			≥9^b^	34	3	42	32	45 (33‐57)	91 (77‐98)	92 (79‐97)	43 (38‐49)
	PNFI + ELF	0.994 (0.917‐0.99)	If PNFI is between 3.47 and 9, use ELF test with a threshold of 8.49	65	4	11	31	86 (76‐93)	89 (73‐97)	94 (62‐83)	74 (62‐83)
Mandelia, 2016	CK‐18 (M30) + waist circumference	0.842 (0.785‐0.900) (validation model 0.839)	≥35^g^	132	40	4	25	97 (93‐99)	38 (27‐51)	77 (73‐80)	86 (69‐95)
			≥82^g^	80	8	56	57	59 (50‐67)	88 (77‐95)	91 (84‐95)	50 (45‐56)
Biomarkers											
Lebensztejn, 2011	CK‐18 (M30)	0.666	≥201 u/L^g^	15	13	4	20	79 (54‐94)	61 (42‐77)	54 (42‐65)	83 (67‐93)
	HA	0.672	≥19.1 ng/mL^g^	16	15	3	18	84 (60‐97)	55 (36‐72)	52 (41‐92)	86 (67‐95)
	HA + CK‐18 (M30)	0.73	HA ≥ 19.1 ng/mL + CK‐18 ≥201 u/L^g^	14	7	5	26	74 (49‐91)	79 (61‐91)	67 (50‐80)	84 (71‐92)
Nobili, 2010	HA	0.88 (0.81‐0.96)	≥1200 ng/mL^h^	40	2	27	31	60 (47‐72)	94 (80‐99)	90 (77‐97)	53 (39‐66)
Nobili, 2009	ELF test	0.92 (0.86‐0.97)	≥9.19	68	13	7	24	91 (82‐96)	65 (47‐80)	84 (77‐89)	77 (62‐88)
			≥9.28^d^	66	7	9	30	88 (78‐94)	81 (65‐92)	90 (83‐95)	77 (64‐86)
			≥9.44	62	4	13	33	83 (72‐90)	89 (75‐97)	94 (86‐98)	72 (60‐81)
Alkhouri, 2011	ELF test	0.924 (0.869‐0.978)	≥8.49^d^	58	1	18	34	76 (65‐85)	97 (85‐100)	98 (89‐100)	65 (56‐74)
			≥9.28	16	0	60	35	21 (13‐32)	100 (90‐100)	100	37 (34‐40)
Imaging techniques											
Schwimmer, 2017	MRE	0.777	≥2.69 kPa^c^ Manual, Centre 2	17	6	19	48	44 (30‐62)	91 (80‐97)	76 (53‐92)	71 (59‐81)
			≥2.77 kPa^c^ Manual, Centre 1	16	5	20	49	44 (28‐62)	91 (80‐97)	76 (53‐92)	71 (59‐81)
			≥2.78 kPa^c^ Automated reading	16	5	20	49	44 (28‐62)	91 (80‐97)	76 (53‐92)	71 (59‐81)
Hudert, 2019	MRE	0.790 (0.664‐0.917)	≥1.46 m/s^d^	25	1	10	14	71 (54‐85)	93 (68‐100)	96 (79‐99)	58 (45‐71)
Nobili, 2008	TE (FibroScan®, M‐probe)	0.977 (90% CI: 0.90‐0.99)	≥5.1 kPa^e^	38	1	1	10	97 (93‐100)	91 (77‐100)	97 (93‐100)	91 (77‐100)
Phelps, 2016	Point SWE (Siemens Acuson S3000)	0.75 (0.33‐1.00)	≥1.7 m/s^f^	3	0	1	1	75 (19‐99)	100 (3‐100)	100	50 (15‐85)
Garcovich, 2017	SWE (Aixplorer US system)	0.924	≥5.1 kPa^d^	42	1	7	18	86 (73‐64)	95 (74‐100)	98 (86‐100)	72 (56‐84)
Hudert, 2018	Time‐harmonic elastography	0.883 (0.804‐0.963)	≥1.52 m/s^d^	37	1	11	18	77 (63‐88)	95 (74‐100)	97 (85‐100)	62 (49‐74)
Farmakis, 2019	SWE (GE LOGIQ E9 system)	0.554 (0.339‐0.768)	≥1.29 m/s^d^	18	8	4	3	83 (60‐95)	27 (6‐61)	69 (60‐77)	43 (17‐74)

Abbreviations: AUC, area under the receiver operating characteristics curve; CK‐18, cytokeratin‐18; ELF test, enhanced liver fibrosis test; FN, false negatives; FP, false positives; HA, hyaluronic acid; MRE, magnetic resonance elastography; NPV, negative predictive value; PNFI, paediatric NAFLD fibrosis index; PPV, positive predictive value; TE, transient elastography; TN, true negatives; TP, true positives.

^a^ Optimal threshold aiming for high sensitivity.

^b^ Optimal threshold aiming for high specificity.

^c^ Highest sensitivity with specificity > 90%.

^d^ Optimal threshold when maximizing sensitivity and specificity/Youden index.

^e^ LR + >10.

^f^ PPV 100%.

^g^ Method for finding optimal threshold not reported.

^h^ Other method for finding optimal threshold.

Among the prediction scores and biomarkers, only the three studies using the ELF test alone or combined with PNFI showed an AUC greater than 0.90. However, the optimal threshold for the ELF test alone as reported by Nobili et al. (9.28)^24^ could not be reproduced by Alkhouri et al. who reported a far lower optimal threshold (8.49).^17^ All other evaluated biomarkers had a lower AUC and reported either a low sensitivity or specificity at their optimal threshold. The PNFI is the only test with accuracy data reported at a wide range of thresholds. Nobili et al. found that a score < 3 could be used to rule out fibrosis with a sensitivity of 96%, and a score of ≥9 could be used to rule in fibrosis with a specificity of 98%.^26^ However, this resulted in 56% of patients with an undetermined classification. Alkhouri et al. validated these thresholds with similar accuracy results and 52% with an undetermined classification.^17^ He subsequently combined the PNFI with the ELF test for patients with an undetermined classification, which resulted in classifying all patients with an overall sensitivity of 86% and specificity of 89%.^17^

Most imaging techniques showed higher accuracy than prediction scores and biomarkers for detecting mild fibrosis. Near perfect accuracy was reported for TE of FibroScan® (AUC 0.98, 95% CI: 0.90‐0.99) resulting in a sensitivity and specificity of both >90% at a threshold of 5.1 kPa in the only study available.^23^

None of the studies validated their results externally. Three studies performed internal cross‐validation using bootstrapping.^22^, ^25^, ^26^ The use of different optimal thresholds reported in the different studies on HA and imaging techniques prevented comparison of accuracy findings among studies in this systematic review. Only the PNFI and ELF test could be compared between studies, yielding discordant results for the optimal threshold in the latter as described above.

### 3.6 Detecting significant fibrosis (≥F2)

Ten studies evaluated the accuracy of non‐invasive methods to detect significant fibrosis (≥F2). These studies addressed the prediction score PFNI, the biomarkers HA, CK‐18 and procollagen type III amino terminal peptide (PIIINP) and the imaging techniques MRE, TE of Fibroscan®, time‐harmonic elastography and SWE. Table 3 presents the results of these ten studies.

**TABLE 3:** Studies on detecting significant fibrosis

Study	Test	AUC (95% CI)	Threshold	TP	FP	FN	TN	Sensitivity (95% CI)	Specificity (95% CI)	PPV (95% CI)	NPV (95% CI)
Prediction scores											
Alkhouri, 2013	PNFI	0.747 (0.632‐0.820)	≥8.2^b^	9	24	1	41	90 (56‐100)	63 (50‐74)	27 (20‐35)	98 (86‐100)
Biomarkers											
Nobili, 2009	ELF test	0.98 (0.96‐1.00)	≥10.09	17	11	0	84	100 (80‐100)	88 (80‐94)	61 (47‐73)	100
			≥10.18^b^	16	7	1	88	94 (71‐100)	93 (85‐97)	70 (53‐83)	99 (93‐100)
			≥10.30	14	0	3	95	82 (57‐96)	100 (96‐100)	100	97 (92‐99)
Nobili, 2010	HA	0.95 (0.91‐0.99)	≥2100 ng/mL^e^					89 (75‐100)	78 (67‐86)	40 (5‐85)	91 (83‐96)
Fitzpatrick, 2010	CK‐18 (M30)	0.66 (CI 0.5‐0.82)	≥200 IU/L^d^	13	19	2	66	83 (61‐95)	41 (21‐64)	60 (50‐68)	69 (45‐86)
Mosca, 2019	PIIINP	0.922 (0.871‐0.972)	≥8.89 ng/mL^b^	38	10	7	149	84 (71‐94)	94 (89‐97)	79 (67‐88)	96 (92‐98)
Imaging techniques											
Nobili, 2008	TE (FibroScan®, M‐probe)	0.992 (90% CI: 0.92‐0.99)	≥7.4 kPa^c^	12	3	0	35	100 (82‐100)	92 (82‐97)	80 (59‐92)	100 (93‐100)
Alkhouri, 2013	TE (FibroScan®, S‐probe)	1.00 (0.98‐1.00)	≥8.6 kPa^b^	10	0	0	57	100 (69‐100)	100 (94‐100)	100	100
	PNFI + TE (FibroScan®, S‐probe)	NR	If is PNFI > 8.2, use TE with a cut‐off of 8.6 kPa	9	0	1	57	90 (56‐100)	100 (94‐100)	100	98 (90‐100)
Trout, 2018	MRE	0.53 (0.35‐0.71)	≥0.94 kPa^a^ From NAFLD subgroup	3	0	20	21	13 (3‐34)	100 (84‐100)	100	51 (47‐55)
			≥1.67 kPa^a^ From total cohort	3	3	20	18	13 (3‐34)	86 (64‐97)	50 (18‐82)	47 (42‐53)
			≥2.27 kPa^b^ From total cohort	12	6	11	15	52 (31‐74)	71 (48‐89)	67 (48‐81)	58 (45‐69)
			≥2.28 kPa^b^ From NAFLD subgroup	12	6	11	15	52 (31‐74)	71 (48‐89)	67 (48‐81)	58 (45‐69)
Hudert, 2019	Multifrequency MRE	0.907 (0.827‐0.986)	≥1.48 m/s^a^	17	2	6	25	74 (52‐90)	93 (76‐99)	89 (69‐97)	81 (68‐89)
Garcovich, 2017	SWE (Aixplorer US system)	0.966	≥6.7 kPa^b^	14	2	2	50	88 (62‐98)	96 (87‐100)	88 (64‐99)	96 (87‐98)
Hudert, 2018	US time‐harmonic elastography	0.994 (0.982‐1.000)	≥1.62 m/s^b^	30	0	1	36	97 (83‐100)	100 (90‐100)	100	97 (84‐100)

Abbreviations: AUC, area under the receiver operating characteristics curve; CK‐18, cytokeratin 18; ELF test, enhanced liver fibrosis test; FN, false negatives; FP, false positives; HA, hyaluronic acid; MRE, magnetic resonance elastography; NPV, negative predictive value; NR, not reported; PIIINP, procollagen type III amino terminal peptide; PNFI, paediatric NAFLD fibrosis index; PPV, positive predictive value; SWE, shear wave elastography; TE, transient elastography; TN, true negatives; TP, true positives.

^a^ Highest sensitivity with specificity > 90%.

^b^ Optimal threshold when maximizing sensitivity and specificity/Youden index.

^c^ LR + >10.

^d^ Method for finding optimal threshold not reported.

^e^ Other method for finding optimal threshold.

Among the biomarkers, the ELF test showed a remarkably high AUC (0.98, 95% CI: 0.96‐1.00) resulting in a sensitivity of 94% and specificity of 93% at a threshold of 10.18 in the only study available.^24^ High AUCs were also reported for two of the three components of the ELF test: HA and PIIINP.^25^, ^30^

TE of FibroScan® was evaluated by Alkhouri et al. and Nobili et al., and both reported an excellent AUC close to 1.00 with a sensitivity of 100% in both studies and a specificity of 92% and 100%, respectively, at slightly different optimal thresholds while using different probes (8.2 kPa with S‐probe^34^ and 7.4 kPa with M‐probe,^23^ respectively). The two evaluated shear wave elastography methods showed high AUCs with high specificity and a sensitivity of >70%.^19^, ^32^ Trout et al. reported an AUC of 0.53 (95% CI: 0.35‐0.71) for MRE using one frequency (60 Hz) for the external vibrations, while Hudert et al. reported an AUC of 0.91 (95% CI: 0.83‐0.99) using seven frequencies (30‐60 Hz).^20^, ^29^

None of the studies used an external validation cohort. Only one study performed internal validation using bootstrapping.^25^ Only for imaging techniques (TE of Fibroscan®, MRE and ultrasound elastography) multiple studies were included; however, pooling of results was not feasible due to the use of different optimal thresholds, FibroScan® probes, MRE settings and ultrasound elastography techniques.

### 3.7 Detecting advanced fibrosis (≥F3)

Nine studies evaluated the accuracy of non‐invasive methods to detect advanced fibrosis (≥F3). These studies addressed the prediction score paediatric NAFLD fibrosis score (PNFS) which is based on alanine aminotransferase (ALT), platelet counts and gamma glutamyl transferase (GGT). The evaluated biomarkers were monocyte chemoattractant protein 1 (MCP‐1), plasminogen activator inhibitor 1 (PAI‐1) and PIIINP, and the imaging techniques were MRE, TE of FibroScan® and time‐harmonic elastography. Table 4 presents the results of these nine studies.

**TABLE 4:** Studies on detecting advanced fibrosis

Study	Test	AUC (95% CI)	Threshold	TP	FP	FN	TN	Sensitivity (95% CI)	Specificity (95% CI)	PPV (95% CI)	NPV (95% CI)
Prediction scores											
Alkhouri, 2014	PNFS	0.74 (0.66‐0.82) (in validation model 0.71)	≥8^a^	35	138	1	68	97 (85‐100)	33 (27‐40)	20 (19‐22)	96 (91‐100)
			≥26^b^	11	16	25	190	31 (16‐48)	92 (88‐96)	41 (26‐58)	88 (86‐90)
Biomarkers											
Nobili, 2009	ELF test	0.99 (0.97‐1.00)	≥10.51^d^	8	2	0	102	100 (63‐100)	98 (93‐100)	80 (50‐94)	100
			≥10.78	4	1	4	103	50 (16‐84)	99 (95‐100)	80 (34‐97)	96 (93‐98)
			≥11.56	2	0	6	104	25 (3‐65)	100 (97‐100)	100	95 (92‐96)
Fitzpatrick, 2012	MCP‐1	0.76 (0.62‐0.91)	≥200 ng/L^g^	12	8	1	19	92 (64‐100)	70 (50‐86)	60 (45‐73)	95 (74‐99)
	PAI‐1	0.78 (0.6‐0.91)	≥52.7 μg/L^g^	11	8	2	19	85 (55‐98)	70 (50‐86)	58 (42‐72)	90 (72‐97)
Lee, 2013	HA	0.729 (0.379‐1.000)	≥43 ng/mL^d^, ^e^	0	0	4	6	0 (0‐60)	100 (54‐100)	0	60 (60‐60)
Mosca, 2019	PIIINP	0.994 (0.984‐1.000)	≥13.2 ng/mL^d^	7	2	0	195	100 (59‐100)	99 (97‐100)	78 (47‐93)	100
Imaging techniques											
Schwimmer, 2017	MRE	0.925	≥3.03 kPa^c^ Manual, Centre 2	2	5	4	79	33 (4‐78)	94 (87‐98)	29 (4‐71)	95 (88‐99)
			≥3.05 kPa^c^ Manual, Centre 1	3	7	3	77	50 (12‐88)	92 (84‐97)	30 (7‐62)	96 (89‐99)
			≥3.33 kPa^c^ Automated reading	2	8	4	76	33 (4‐78)	91 (82‐96)	20 (3‐56)	95 (88‐99)
Hudert, 2019	MRE	0.895 (0.802‐0.988)	≥1.53 m/s^c^	9	3	5	33	64 (35‐87)	92 (78‐98)	75 (49‐90)	87 (76‐93)
Lee, 2013	TE (FibroScan®, M‐probe)	0.625 (0.188‐1.000)	≥8.6 kPa^d^, ^e^	2	1	2	3	50 (7‐93)	75 (19‐99)	67 (22‐93)	60 (33‐82)
Nobili, 2008	TE (FibroScan® M‐probe)	1.00 (90% CI: 0.94‐1.00)	≥10.2 kPa^f^	5	0	0	45	100 (65‐100)	100 (94‐100)	100 (65‐100)	100 (94‐100)
Hudert, 2018	Time‐harmonic elastography	0.880 (0.800‐0.960)	≥1.64 m/s^d^	18	10	0	39	100 (81‐100)	80 (66‐90)	64 (51‐76)	100

Abbreviations: AUC, area under the receiver operating characteristics curve; ELF test, enhanced liver fibrosis test; FN, false negatives; FP, false positives; HA, hyaluronic acid; MCP‐1, monocyte chemoattractant protein 1; MRE, magnetic resonance elastography; NPV, negative predictive value; PAI, plasminogen activator inhibitor 1; PIIINP, procollagen type III amino terminal peptide; PNFS, paediatric NAFLD fibrosis score; PPV, positive predictive value; TE, transient elastography; TN, true negatives; TP, true positives.

^a^ Optimal threshold aiming for high sensitivity.

^b^ Optimal threshold aiming for high specificity.

^c^ Highest sensitivity with specificity > 90%.

^d^ Optimal threshold when maximizing sensitivity and specificity/Youden index.

^e^ Optimal threshold found in cohort of children with various liver diseases.

^f^ LR + >10.

^g^ Method for finding optimal threshold not reported.

Again, among the biomarkers, the ELF test showed a near perfect accuracy (AUC 0.99, 95% CI: 0.97‐1.00) in the only included study by Nobili et al.^24^ Its component PIIINP had an equally near perfect AUC of 0.99 (95% CI: 0.98‐1.00),^30^ while HA, another component of the ELF test, showed a mediocre AUC of 0.73 (95% CI: 0.38‐1.00).^37^ All other prediction scores and biomarkers had AUCs ranging between 0.71 and 0.78, and only single studies were included for each. MRE was evaluated in two studies that both showed good AUCs, but this did not result in a high sensitivity when aiming for a specificity of >90%.^20^, ^28^ The perfect accuracy reported by Nobili et al. for TE of FibroScan^®^ ^23^ could not be reproduced by Lee et al.^37^ None of the studies used an external validation cohort. Only one study performed internal validation using bootstrapping.^35^ Lee et al. used a calibration and validation cohort, although this was applied to the entire cohort of children with various liver disease and not to the subgroup of ten children with NAFLD for which we received raw data.^37^ Only for the imaging techniques MRE and TE of FibroScan®, multiple studies were included. However, the use of different optimal thresholds precluded pooling their results.

## 4 DISCUSSION

To the best of our knowledge, this is the first systematic review on the diagnostic accuracy of fibrosis tests in paediatric NAFLD. We reported the accuracy of fibrosis tests in detecting mild, significant or advanced fibrosis in children with NAFLD using histology as a reference standard. Diagnostic accuracy was determined in 20 studies encompassing a total of 1787 subjects with NAFLD. This systematic review shows that although a wide range of fibrosis tests have been studied in children, robust accuracy data are scarce.

Most included studies had a small sample size; only three studies included more than 200 subjects. Out of the 16 fibrosis tests included in this SR, only two tests (PNFI and ELF) had accuracy data for detecting mild fibrosis in two studies with similar thresholds, validating results for this fibrosis stage. However, the generalizability of these results is still compromised because all studies were performed in the same tertiary liver clinic. In addition, some overlap between cohorts in the studies that evaluated the PNFI cannot be excluded because inclusion periods overlapped.^26^, ^34^ For all other tests and fibrosis stages, no external validation was performed. Comparing the accuracy results of serum biomarkers can be complicated by the use of kits from different manufacturers. In this review, this applies to HA, probably explaining the remarkable difference in thresholds between the three studies.^21^, ^25^, ^37^ For imaging techniques, comparing accuracy results among studies was further precluded by the use of different techniques and machines. Despite some studies reporting (near) perfect accuracy, due to the virtually complete lack of validation data, the actual accuracy and clinical utility of all fibrosis tests remains uncertain.

The wide range of optimal thresholds reported by the included studies is explained by the high variety in methods to determine this threshold. These include, among others, the Youden index, optimizing sensitivity or specificity, a PPV of 100% and a positive likelihood ratio > 10. Several studies did not report how the optimal threshold was defined or determined it in a cohort of children with various liver diseases. The wide range of reported thresholds shows the lack of consensus on the minimal diagnostic performance of fibrosis tests in NAFLD, while also highlighting the need to report operating characteristics curves and to publish raw study data. In 2018, the LITMUS consortium (Liver Investigation: Testing Marker Utility in Steatohepatitis) published a report describing the minimal acceptable performance criteria in the different possible contexts of use of biomarkers in adult NAFLD (e.g. screening, diagnosing, prognostic and monitoring).^38^ Although not aimed at children, these context and criteria are equally relevant in paediatric NAFLD. Future biomarker studies should be designed and reported taking into account applicability and minimal acceptable performance criteria for its intended context of use of the biomarker.

Applicability of the reported study results is compromised by the fact that all included studies were performed in tertiary liver centres, half of those studies originate from the same European centre, and all were performed in severely metabolically affected children who underwent liver biopsy based on clinical grounds. Although the latter is inevitable due to ethical restraints when performing a liver biopsy, it is important to take them into account when considering the applicability of the liver fibrosis tests in other settings, particularly in a screening setting.

A strength of this study is the sensitive and well‐defined search strategy that lowered the risk of missing relevant studies. As we anticipated for a low prevalence of fibrosis, we included studies with at least 60 children, to provide a more solid reflection of the test accuracy. However, a lower number of patients was accepted if the index test was evaluated in at least two studies. Included studies with <60 participants evaluated MRE,^20^, ^29^ SWE,^18^, ^27^ TE,^23^, ^37^ HA^21^, ^37^ and CK‐18.^21^, ^33^ A limitation of this SR is that summary AUCs or summary sensitivities and specificities could not be calculated for any fibrosis test based on the published data and we did not collect raw data of all included studies.

In conclusion, the available data on the accuracy of non‐invasive fibrosis tests in children with NAFLD are insufficient to determine their accuracy. As studies have solely been performed in tertiary care settings, accuracy data cannot directly be translated to screening populations. The most promising tests identified are TE by FibroScan®, ELF test and ultrasound SWE, as they were evaluated in more than one study and showed consistent good performance. Future studies are needed to validate the most promising tests and determine their accuracy in different clinical settings. Future studies should report diagnostic accuracy over the full range of fibrosis grades using standardized outcomes including receiver operating characteristics curves discuss the applicability of the results for its context of use and relate to the minimally acceptable performance of the biomarker for that context as defined by the LITMUS consortium.^38^ Collaborations between paediatric centres on an international level, such as the NASH CRN and the paediatric European NAFLD registry, are needed to create a large diverse cohort with well‐characterized children with NAFLD.^39^, ^40^

## REGISTRATION

The protocol of this systematic review is available in PROSPERO: CRD42019117504.

## PATIENT CONSENT

Not applicable for systematic review.

## CONFLICT OF INTEREST

All authors do not have any disclosures to report.

## ETHICS APPROVAL

Not applicable for systematic review.

## Supporting information

Data S1

Data S2

Data S3

Data S4
