# Supplementary material for: Diagnostic accuracy of fibrosis tests in children with non‐alcoholic fatty liver disease: A systematic review
Source: Liver Int. 2021 May 11;41(9):2087–100. doi: 10.1111/liv.14908 (PMC8453517; doi:10.1111/liv.14908)
Supplement: Supplementary file 1 — Data S1 [file LIV-41-2087-s001.docx]

**Supplemental File 1. Search strategies**

PUBMED

("Non-alcoholic Fatty Liver Disease"[Mesh] OR "Fatty Liver"[Mesh] OR "Liver/diagnostic imaging"[Mesh] OR "Liver/diagnosis"[Mesh] OR non-alcoholic fatty liver*[tiab] OR nonalcoholic fatty liver*[tiab] OR NAFLD[tiab] OR nonalcoholic steatohepatiti*[tiab] OR non-alcoholic steatohepatiti*[tiab] OR steatosis[tiab] OR steatoses[tiab] OR fatty liver[tiab]) AND ("Child"[Mesh] OR "Child, Preschool"[Mesh] OR "Infant"[Mesh] OR "Infant, Newborn"[Mesh] OR "Infant, Low Birth Weight"[Mesh] OR "Infant, Postmature"[Mesh] OR "Infant, Premature"[Mesh] OR "Adolescent"[Mesh] OR "Pediatrics"[Mesh] OR child*[tiab] OR infan*[tiab] OR newborn*[tiab] OR neonat*[tiab] OR baby[tiab] OR babies[tiab] OR pediatric*[tiab] OR paediatric*[tiab] OR adoles*[tiab] OR teen*[tiab] OR youth*[tiab] OR schoolchild*[tiab] OR preschool[tiab] OR pre-school[tiab] OR kid[tiab] OR kids[tiab] OR toddler*[tiab] OR juvenil*[tiab] OR teen*[tiab] OR under*age* OR pubescen*[tiab] OR puber*[tiab] OR prepubert*[tiab] OR school age*[tiab] OR schoolage*[tiab] OR elementary school[tiab] OR high school*[tiab] OR highschool*[tiab] OR kindergar*[tiab] OR boy[tiab] OR boys[tiab] OR girl*[tiab] OR minor*[tiab] OR underag*[tiab] OR under ag*[tiab]) AND

("Biomarkers"[Mesh] OR "Diagnosis"[Mesh] OR "Sensitivity and Specificity"[Mesh] OR "Reproducibility of Results"[Mesh] OR "Elasticity Imaging Techniques"[Mesh] OR "diagnosis" [Subheading] OR "diagnostic imaging" [Subheading] OR biomarker*[tiab] OR marker*[tiab] OR predict*[tiab] OR score*[tiab] OR diagnos*[tiab] OR accura*[tiab] OR valid*[tiab] OR sensitiv*[tiab] OR specific*[tiab] OR elastogra*[tiab] OR fibroscan*[tiab] OR fibro- scan*[tiab]  OR shear wave*[tiab] OR ARFI[tiab] OR imaging[tiab] OR predict*[tiab] OR ROC-curve[tiab] OR receiver-operator*[tiab] OR likelihood[tiab] OR inter-observer[tiab] OR intra-observer[tiab] OR kappa[tiab] OR reliability[tiab] OR reproducibility[tiab] OR fibrotest[tiab] OR AAR[tiab]  OR  APRI[tiab] OR ELF test[tiab] OR PNFI[tiab] OR pediatric nafld fibrosis index[tiab] OR  PNFS[tiab] OR pediatric nafld fibrosis score*[tiab] OR paediatric nafld fibrosis score*[tiab] OR  MR[tiab] OR  MR-elastograph*[tiab] OR  ultraso*[tiab] OR sonograph*[tiab]) AND ("Fibrosis"[Mesh] OR "Severity of Illness Index"[Mesh] OR fibros*[tiab] OR severity[tiab] OR liver stiffness*[tiab])

EMBASE (Ovid)

| # | Searches | Results |
| --- | --- | --- |
| 1 | nonalcoholic fatty liver/ or fatty liver/ or (non-alcoholic fatty liver* or nonalcoholic fatty liver* or NAFLD or nonalcoholic steatohepatiti* or non-alcoholic steatohepatiti* or steatosis or steatoses or fatty liver).ti,ab,kw. | 74644 |
| 2 | child/ or preschool child/ or infant/ or newborn/ or adolescent/ or exp low birth weight/ or postmaturity/ or prematurity/ or exp pediatrics/ or (child* or infant* or infancy or newborn* or neonat* or baby or babies or pubescen* or teen* or adolescen* or puber* or prepubert* or juvenil* or p?ediatric* or youth* or schoolchild* or school age* or schoolage* or preschool or pre-school or elementary school or high school* or highschool* or kindergar* or boy or boys or girl* or minor* or underag* or under ag* or kid or kids or toddler*).ti,ab,kw. | 4681177 |
| 3 | biological marker/ or marker/ or exp diagnosis/ or exp "sensitivity and specificity"/ or reproducibility/ or exp elastography/ or diagnosis.fs. or exp diagnosis/ or exp "sensitivity and specificity"/ or reproducibility/ or exp elastography/ or diagnosis.fs. or (biomarker* or marker* or predict* or score* or diagnos* or accura* or valid* or sensitiv* or specific* or elastogra* or fibroscan* or fibro- scan* or shear wave* or ARFI or imaging or predict* or ROC-curve or receiver-operator* or likelihood or inter-observer or intra-observer or kappa or reliability or reproducibility or fibrotest or AAR or APRI or ELF test or PNFI or pediatric nafld fibrosis index or PNFS or pediatric nafld fibrosis score* or paediatric nafld fibrosis score* or MR or MR-elastograph* or ultraso* or sonograph*).ti,ab,kw. | 14681095 |
| 4 | exp fibrosis/ or "severity of illness index"/ or (fibros* or severity or liver stiffness*).ti,ab,kw. | 948253 |
| 5 | 1 and 2 and 3 and 4 | 1964 |
| 6 | limit 5 to conference abstract status | 744 |
| 7 | 5 not 6 | 1220 |


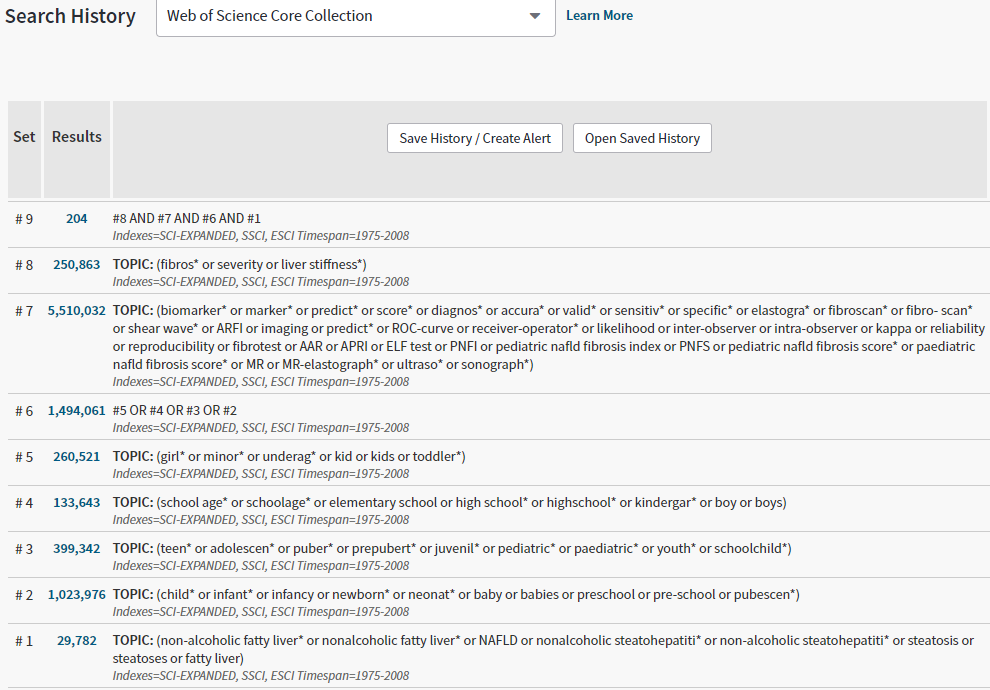
WEB OF SCIENCE
