# Supplementary material for: Diagnostic accuracy of fibrosis tests in children with non‐alcoholic fatty liver disease: A systematic review
Source: Liver Int. 2021 May 11;41(9):2087–100. doi: 10.1111/liv.14908 (PMC8453517; doi:10.1111/liv.14908)
Supplement: Supplementary file 2 — Data S2 [file LIV-41-2087-s002.pdf]

Cochrane Library

Access provided by: **Universiteit van Amsterdam**

Advanced Search

Please note that the Advanced Search is optimised for English search terms. Certain features, such as search operators and MeSH terms, are only available in English.

Search manager

Save this search | View saved searches | Search help

View fewer lines | Print

#1	MeSH descriptor: [Non-alcoholic Fatty Liver Disease] explode all trees	MeSH	559
#2	MeSH descriptor: [Fatty Liver] explode all trees	MeSH	848
#3	MeSH descriptor: [Liver] explode all trees and with qualifier(s): [diagnostic imaging - DG]	MeSH	237
#4	(non-alcoholic fatty liver* or nonalcoholic fatty liver* or NAFLD or nonalcoholic steatohepatiti* or non-alcoholic steatohepatiti* or steatosis or steatoses or fatty liver):ti,ab,kw	S	3169
#5	#1 or #2 or #3 or #4		3372
#6	MeSH descriptor: [Child] explode all trees	MeSH	1403
#7	MeSH descriptor: [Infant] explode all trees	MeSH	14988
#8	MeSH descriptor: [Adolescent] explode all trees	MeSH	97405
#9	(child* or infant* or infancy or newborn* or neonat* or baby or babies or preschool or pre school or pubescen* or teen* or adolescen* or puber* or prepubert* or juvenil* or p?ediatric* or youth* or schoolchild* or school age* or schoolage* or preschool or pre-school or elementary school or high school* or highschool* or kindergar* or boy or boys or girl* or minor* or underag* or under ag* or kid or kids	S	287251
#10	#6 or #7 or #8 or #9		287251
#11	MeSH descriptor: [Biomarkers] explode all trees	MeSH	18303
#12	MeSH descriptor: [Diagnosis] explode all trees	MeSH	305878

− +	#13	MeSH descriptor: [Sensitivity and Specificity] explode all trees	MeSH		15772
− +	#14	MeSH descriptor: [Reproducibility of Results] explode all trees	MeSH		11221
− +	#15	MeSH descriptor: [Elasticity Imaging Techniques] explode all trees	MeSH		156
− +	#16	(biomarker* or marker* or predict* or score* or diagnos* or accura* or valid* or sensitiv* or specific* or elastogra* or fibroscan* or fibro scan* or shear wave* or ARFI or imaging or predict* or ROC curve or receiver operator* or likelihood or inter observer or intra observer or kappa or reliability or reproducibility or fibrotest or AAR or APRI or ELF test or PNFI or pediatric nafld fibrosis index or PNFS or pediatric nafld fibrosis score* or paediatric nafld fibrosis score* or MR or ultraso* or sonograph*):ti,ab,kw	S		495668
− +	#17	#11 or #12 or #13 or #14 or #15 or #16			635524
− +	#18	MeSH descriptor: [Fibrosis] explode all trees	MeSH		2390
− +	#19	MeSH descriptor: [Severity of Illness Index] explode all trees	MeSH		18439
− +	#20	(fibros* or severity or liver stiffness*):ti,ab,kw	S		82846
− +	#21	#18 or #19 or #20			84517
− +	#22	#5 and #10 and #17 and #21			150
− +	#23	Manually type a search term here or click on the S	S	MeSH	N/A

✖ Clear all

Highlight orphan lines

Save this search | View saved searches | ? Search help

View fewer lines | Print

Filter your results

Cochrane Reviews 1 | Cochrane Protocols 0 | Trials 149 | Editorials 0 | Special collections 0 | Clinical 0 | More

Other Reviews

1 Cochrane Review matching on '"#22 - #5 and #10 and #17 and #21"'

Did you mean: *Rand band hand*

Cochrane Database of Systematic Reviews

Issue 9 of 12, September 2018

Select all (1) Export selected citation(s) Show all previews

Order by Relevancy Results per page 25

1

Antioxidant supplements for non-alcoholic fatty liver disease and/or steatohepatitis

Flavio Lirussi, Lorenzo Azzalini, Serena Orando, Rocco Orlando, Francesco Angelico

Show Preview Intervention Review 24 January 2007

Show Preview
