# Supplementary material for: Diagnostic accuracy of fibrosis tests in children with non‐alcoholic fatty liver disease: A systematic review
Source: Liver Int. 2021 May 11;41(9):2087–100. doi: 10.1111/liv.14908 (PMC8453517; doi:10.1111/liv.14908)
Supplement: Supplementary file 3 — Data S3 [file LIV-41-2087-s004.docx]

## **Supplementary File 2.** Scoring systems for liver fibrosis

| **Fibrosis**  **Distribution** | **NASH CRN fibrosis stage(1)** | **Ishak**  **Fibrosis stage(2)** | **METAVIR fibrosis stage(3)** |
| --- | --- | --- | --- |
| No excess fibrosis | F0 | F0 | F0 |
| Portal or perisinusoidal | F1 | F1 | F1 |
| Portal and perisinusoidal | F2 | F2 | F1 |
| Bridging | F3 | F3-4 | F2-3 |
| Nodules | F4 | F5- 6 | F4 |

Abbreviations: NASH CRN, nonalcoholic steatohepatitis clinical research network

References:

1. Kleiner DE, Brunt EM, Van Natta M, Behling C, Contos MJ, Cummings OW, et al. Design and validation of a histological scoring system for nonalcoholic fatty liver disease. Hepatology. 2005;41(6):1313-21.

2. Ishak K, Baptista A, Bianchi L, Callea F, De Groote J, Gudat F, et al. Histological grading and staging of chronic hepatitis. J Hepatol. 1995;22(6):696-9.

3. Intraobserver and interobserver variations in liver biopsy interpretation in patients with chronic hepatitis C. The French METAVIR Cooperative Study Group. Hepatology. 1994;20(1 Pt 1):15-20.
