# Supplementary material for: Diagnostic accuracy of fibrosis tests in children with non‐alcoholic fatty liver disease: A systematic review
Source: Liver Int. 2021 May 11;41(9):2087–100. doi: 10.1111/liv.14908 (PMC8453517; doi:10.1111/liv.14908)
Supplement: Supplementary file 4 — Data S4 [file LIV-41-2087-s003.docx]

| **Study** | **Index test** | **Target condition** | **AUC (95% CI)** |
| --- | --- | --- | --- |
| **Prediction scores** | | | |
| Alkhouri, 2011^σ^ | PNFI | ≥F2 | 0.663 (0.488–0.837) |
|  |  | ≥F3 | 0.618 (0.397–0.839) |
| Yang, 2012 | FIB-4 Index | ≥F2 | 0.81 (0.68-0.94) |
|  | Forn Index | ≥F2 | 0.73 (0.58-0.88) |
|  | NAFLD Fibrosis Score | ≥F2 | 0.58 (0.41-0.75) |
|  | PGA score | ≥F2 | 0.45 (0.28-0.62) |
|  | PNFI | ≥F2 | 0.41 (0.24-0.58) |
| Alkhouri, 2014^σ^ | FIB-4 Index | ≥F3 | 0.64 |
|  | NAFLD Fibrosis Score | ≥F3 | 0.60 |
| Mansoor, 2015 | Fib-4 Index | ≥F1 | 0.547 (0.375-0.719) |
|  |  | ≥F2 | 0.686 (0.576-0.797) |
|  |  | ≥F3 | 0.367 (0.231-0.503) |
|  | NAFLD Fibrosis Score | ≥F1 | 0.470 (0.259-0.681) |
|  |  | ≥F2 | 0.554 (0.435-0.673) |
|  |  | ≥F3 | 0.521 (0.385-0.657) |
| Jackson, 2018 | FIB-4 | ≥F1 | 0.59 (0.475-0.703) |
|  | PNFI | ≥F1 | 0.67 (0.435-0.898) |
|  | PNFS | ≥F1 | 0.57 (0.401-0.729) |
| Mosca, 2019 ^σ^ | FIB-4 Index | ≥F2 | 0.741 |
|  |  | ≥F3 | 0.769 |
| **Biomarkers** | | | |
| Patton, 2008 | AST | ≥F1 | 0.61 |
|  |  | ≥F2 | 0.66 |
|  |  | ≥F3 | 0.74 |
|  | ALT | ≥F1 | 0.55 |
|  |  | ≥F2 | 0.62 |
|  |  | ≥F3 | 0.69 |
|  | AST + ALT | ≥F1 | 0.66 |
|  |  | ≥F2 | 0.65 |
|  |  | ≥F3 | 0.74 |
| Alkhouri, 2011^σ^ | ELF Test | ≥F2 | 0.968 (0.937–0.998) |
|  |  | ≥F3 | 0.962 (0.925–0.998) |
| Yang, 2012 | APRI | ≥F2 | 0.70 (0.55-0.86) |
|  | AST/ALT ratio | ≥F2 | 0.53 (0.35-0.70) |
| Alkhouri, 2014^σ^ | APRI | ≥F3 | 0.67 |
| Mandelia, 2016^σ^ | CK-18 (M30) | ≥F1 | 0.75 (0.68-0.81) |
|  |  | ≥F2 | 0.67 (0.54-0.80) |
|  |  | ≥F3 | 0.77 (0.56-0.97) |
| Mansoor, 2015 | AST/ALT | ≥F1 | 0.572 (0.350-0.793) |
|  |  | ≥F2 | 0.585 (0.466-0.703) |
|  |  | ≥F3 | 0.441 (0.316-0.565) |
|  | APRI | ≥F1 | 0.800 (0.695-0.904) |
|  |  | ≥F2 | 0.666 (0.553-0.778) |
|  |  | ≥F3 | 0.628 (0.478-0.778) |
| Jackson, 2018 | ALT | ≥F1 | 0.64 (0.540-0.731) |
|  | AST | ≥F1 | 0.64 (0.544-0.738) |
|  | AST/ALT ratio | ≥F1 | 0.51 (0.406-0.623) |
|  | APRI | ≥F1 | 0.67 (0.556-0.775) |
| Mosca, 2019^σ^ | APRI | ≥F2 | 0.766 |
|  |  | ≥F3 | 0.854 |
| **Radiological methods** | | | |
| Fitzpatrick, 2013* | TE (FibroScan®, M-probe) | ≥F2 | 0.73 |
|  |  | ≥F3 | 0.80 |
| Dillman, 2019 | MRE | ≥F2 | 0.774 |

Supplemental File 3. Studies that provided only AUCs for detecting mild, significant or advanced fibrosis.

Abbreviations: ALT, alanine aminotransferase; APRI, AST to platelet ratio index; AST, aspartate aminotransferase; AUC, area under the receiver operating characteristics curve; CK-18, cytokeratin-18; ELF test, enhanced liver fibrosis test; FIB-4, fibrosis-4; MRE, magnetic resonance elastography; NAFLD, Non-alcoholic fatty liver disease; PNFI, Pediatric NAFLD Fibrosis Index; PGA, Prothrombin time, gamma-glutamyl transpeptidase, apolipoprotein A1; PNFS, pediatric NAFLD fibrosis score; TE, transient elastography

* Fitzpatrick et al. reported an optimal cutoff of 6.1 kPa for detecting ≥F2 fibrosis with a sensitivity of 60% and a specificity of 78%; and an optimal cutoff of 6.9 kPa for detecting ≥F3 fibrosis with a sensitivity of 72% and a specificity of 85%. As the number of patients per fibrosis stage was not reported, it was not possible to create a 2x2 table.
^σ^ Also included in the analysis (Table 2-4) but reports separately on other target conditions using only AUCs.
